# Improving the Energy Saving Process with High-Resolution Data: A Case Study in a University Building

**DOI:** 10.3390/s18051606

**Published:** 2018-05-17

**Authors:** Jeongyun Han, Eunjung Lee, Hyunghun Cho, Yoonjin Yoon, Hyoseop Lee, Wonjong Rhee

**Affiliations:** 1 Department of Transdisciplinary Studies, Seoul National University, Seoul 08826, Korea; hanjy@snu.ac.kr (J.H.); ej-lee@snu.ac.kr (E.L.); webofthink@snu.ac.kr (H.C.); 2 Department of Civil and Environmental Engineering, Korea Advanced Institute of Science and Technology, Daejeon 34141, Korea; yoonjin@kaist.ac.kr; 3 Encored Technologies Inc., Seoul 06109, Korea; hslee@encoredtech.com

**Keywords:** energy saving, data-driven, intervention design, behavior detection, data resolution

## Abstract

In this paper, we provide findings from an energy saving experiment in a university building, where an IoT platform with 1 Hz sampling sensors was deployed to collect electric power consumption data. The experiment was a reward setup with daily feedback delivered by an energy delegate for one week, and energy saving of 25.4% was achieved during the experiment. Post-experiment sustainability, defined as 10% or more of energy saving, was also accomplished for 44 days without any further intervention efforts. The saving was possible mainly because of the data-driven intervention designs with high-resolution data in terms of sampling frequency and number of sensors, and the high-resolution data turned out to be pivotal for an effective waste behavior investigation. While the quantitative result was encouraging, we also noticed many uncontrollable factors, such as exams, papers due, office allocation shuffling, graduation, and new-comers, that affected the result in the campus environment. To confirm that the quantitative result was due to behavior changes, rather than uncontrollable factors, we developed several data-driven behavior detection measures. With these measures, it was possible to analyze behavioral changes, as opposed to simply analyzing quantitative fluctuations. Overall, we conclude that the space-time resolution of data can be crucial for energy saving, and potentially for many other data-driven energy applications.

## 1. Introduction

With the advent of the IoT (Internet Of Things) era, sensor data is becoming rapidly affordable and increasingly influential in the energy field [1]. In particular, energy consumption data has become an integral part of solving a variety of energy problems. For instance, energy consumption data is being used for load prediction [2], population segmentation [3], real-time dashboard [4,5], individual feedback [6], occupancy detection [7], and NILM (Non-Intrusive Load Monitoring) [8]. For the data to be effective in such applications, high data quality is required, where quality can be defined as the resolution in the time and space dimensions [1]. Data resolution directly affects the amount of information that is captured in the data, and therefore determines the level of analysis that can be performed [9]. Collecting high-resolution data, however, implies a higher cost in terms of sensors and data management systems. This results in a tradeoff between data quality and cost, and the optimal resolution is known to be dependent on the task and the final goal [10]. To study the impact of high-quality data on well-known energy problems, we consider the task of energy saving, which has been heavily investigated in recent decades. Most existing studies have focused only on the amount of power consumption, but we are more concerned with the data quality and its impact. Additionally, some studies have incorporated occupancy data collected from other types of sensors [11,12], but we consider only electrical power consumption data in order to keep the study focused on data quality (in fact, we tried installing a few different types of occupancy detection sensors, but encountered accuracy and/or privacy issues in the campus environment. In order to focus on the main topic of this work, occupancy data was excluded in the study).

**Figure 1:**
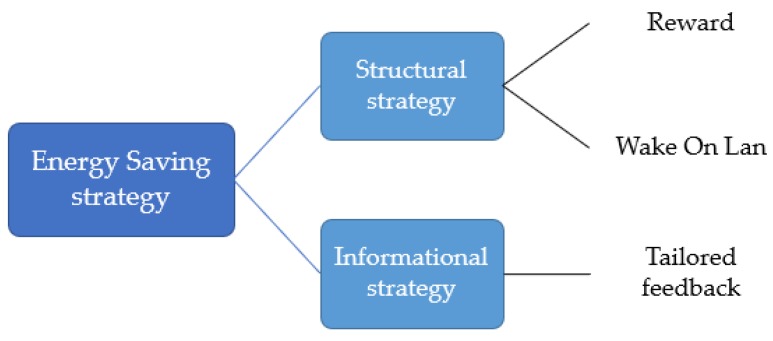
The overall interventions for this study.

Energy saving strategies for encouraging pro-environmental behavior can be divided into two broad categories: structural strategies and informational strategies [13]. According to [13], structural strategies are aimed at making changes in the circumstances under which behavioral choices are made (e.g., improvement of home insulation, rewards, etc.). Typically, a structural change requires a financial investment. Informational strategies are aimed at changing perceptions, motivations, knowledge, and norms, without changing the external context in which choices are made (e.g., periodic emails with recommended behavior changes, consulting, etc.). In general, combining both strategies will be most effective, because there is often more than one barrier inhibiting users from acting pro-environmentally [13,14]. In this study, we utilize data-driven approaches and combine structural strategies and informational strategies. As structural strategies, we provide a reward and use a low-cost system that encourages users to turn off their computers (Wake On Lan; details in Section 4). For informational strategies, data-driven analysis is employed to provide highly tailored feedback, which is known to be effective [15,16,17,18]. The overall interventions for this study are summarized in Figure 1.

Our experiment was performed in a university building. The importance of energy saving in the residential sector has been well recognized, and there have been numerous experiments and studies using interventions such as feedback, reward, and consulting [19,20]. The commercial sector, including university buildings, is also important, and the sector is continuing to grow. For instance, the world’s commercial sector, including education, government, private and public organizations, is expected to be the fastest-growing demand sector with an average energy consumption growth of 1.6% per year between 2012 and 2040 [21], and the service sector accounts for 30% of the total electricity usage in EU [22]. Within the commercial sector, energy consumption in educational buildings was the third highest in the US, according to [23]. A variety of studies have been performed on energy saving in the commercial sector, for instance, see [24,25,26], and a review on data science techniques applied to building energy management can be found in [27].

**Figure 2:**
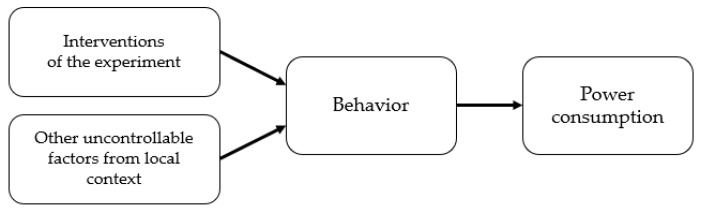
Experiment interventions and uncontrollable factors that affect behavior and power consumption.

There are several challenges for energy behavior research on campus. Students, faculties, and staff on campus are typically not interested in reducing energy usage. Almost no one is aware of the energy bill, and the majority usually behave as if the energy used on campus is free. In particular, students do not realize that the charge is included in their tuition fees [28,29]. Due to the lack of information and interest, it is difficult to motivate people to change their behavior. Even if they can be motivated, it is usually difficult to expect a large saving, because the group does not know what particular energy saving behavior is required and allowed within their local context. Even though it is important to reduce standby power when there is no activity, many devices are shared and used, and energy is wasted because conservation maintenance is often not performed properly [30,31]. Another challenge lies in uncontrollable factors such as exams, papers due, office allocation shuffling, and graduation in a campus environment. User behavior that influences power consumption is affected not only by the interventions but also by the uncontrollable factors (Figure 2). A large-scale experiment is typically needed to cope with such uncontrollable factors. Establishing a large-sized experimental group, however, is usually not feasible in the campus context. Consequently, it becomes difficult to confirm whether a reduction in energy consumption was achieved due to the interventions in the experiment. To resolve this problem, we investigate how high-resolution data can be utilized to mitigate this issue. In particular, we establish methods for utilizing high-quality data to detect energy saving behaviors with high confidence.

The rest of this paper is organized as follows. Section 2 presents the experiment setup. Section 3 explains the space-time resolution of data and how they can be used for detection of energy saving behavior. Section 4 describes the experiment, and Section 5 explains the energy saving results, including power consumption analysis and energy saving behavior analysis. Section 6 supplies discussions on several matters, and Section 7 contains the conclusions.

## 2. Experiment Setup

An IoT data collection system was deployed on the 4th floor of a graduate school building, where three open-space offices (15 m × 6 m) were occupied by graduate students. Each office accommodated between 15 and 20 graduate students, and most of the students took 3~9 h of classes per week with their research topics in the interdisciplinary areas related to computer science or information science. The number of occupants at a given time fluctuated, not only because of unpredictable factors such as exams, papers due, office allocation shuffling, graduation, and newcomers, but also because of the nature of graduate students’ lifestyles. In this work, we chose one of the offices for the experiment. The students in the office were heavy computer users, and some students used 2 or more computers, while most students used two monitors. A summary of pre-experiment consumption measurements is shown in Table 1.

### 2.1. Data Collection

**Figure 3:**
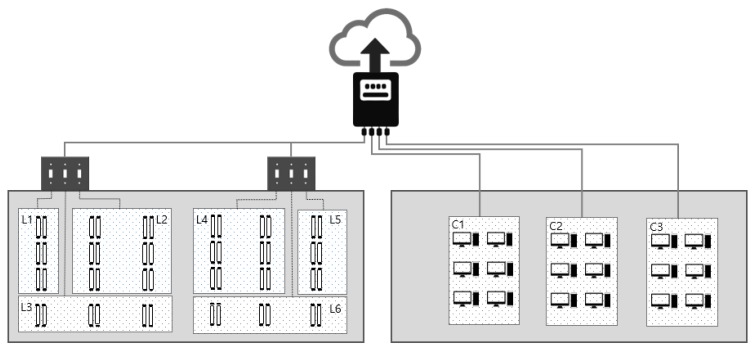
Left side shows the ceiling lights and their switching groups. All six groups were collectively monitored using a single CT sensor. Right side shows the three computer groups. Three CTs were used to monitor each computer group.

In the building, an electricity distribution board existed in each office, and CT (Current Transformer) sensors were deployed on the board. The collected data were sent to a cloud server every second through a LAN connection. Both spatial and temporal resolutions of the collected data were high. Spatial resolution is explained first. The ceiling-side and floor-side sensor configurations are shown in Figure 3. On the ceiling side, the lights were connected to 6 switches, and their mappings can be seen in the figure (L1~L6). The total electricity used by all six light groups was measured by a single CT sensor. On the floor side, power outlets for computers and other appliances were available, and the wirings were grouped into three during the building construction. Therefore, the three groups (C1~C3) were measured using three sensors. The outlets were mainly used for computers and monitors, and one of the outlets had a smaller number of computers and monitors connected compared to the others. In this study, however, we aggregated the electricity usage over the three outlets and used the aggregate as a single computer group for all of the analyses. Energy consumption of HVAC was measured separately, but an independent in-building air-conditioning was also available, and thus HVAC is not considered in this paper. Other outlets were measured, as well (‘Others’ in Table 1), but they are not analyzed in this study because their contribution to the total was relatively small. In summary, one sensor for lights and three sensors for computers were used for each office in order to increase the spatial resolution. As for the time resolution of the data, electricity was measured once per second (1 Hz-sampling) by the sensors. In fact, an even faster sampling based on a 15 Hz collection was made available later, but this study is limited to 1-s and 15-min resolution data. As will be explained in the next section, 15 min was a sufficient resolution for some of the analyses, while 1 s was required for the others.

For the collected data, a few basic methods of data preprocessing were applied before analysis. Missing, incomplete, or abnormal measurement samples were rare, but they had to be properly addressed before applying data analysis functions. This definition is necessary, because graduate students often stayed in the office until 2~3 a.m., but almost never showed up before 7 a.m.

### 2.2. Timeline

The IoT platform for collecting high-quality energy data was deployed on 4 August of 2014, and the experiment was completed in 25 weeks. The timeline of the experiments is summarized in Table 2. The intervention was applied for a total of 2 weeks, where one week of intervention was applied for both the first experiment, E1, and the second experiment, E2. The rest of the experiment periods were used for the pre-experiment baseline calculation and for the post-experiment sustainability analysis.

## 3. Space-Time Resolution and Behavior Detection

To facilitate data-driven intervention design of energy saving, waste behavior investigation using E1-Pre data was performed prior to the intervention experiment E1. Because the deployed IoT system collected 1 Hz data over multiple sensors, we were able to identify frequently occurring wasting behaviors without any human interactions such as surveys or interviews. In this section, the findings are explained with careful attention to the required space-time resolution for each specific finding.

**Figure 4:**
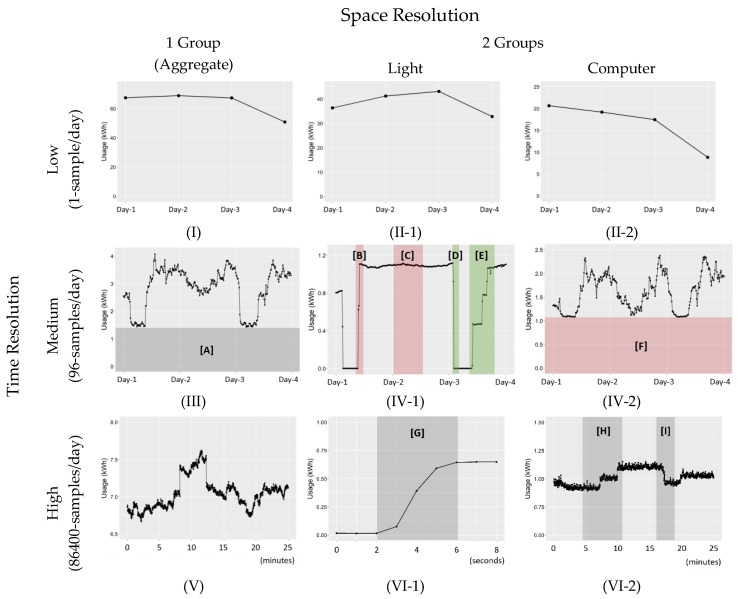
Power consumption plot and behavior detection based on data patterns (sample intervals from the trial data). Combinations of three time resolution levels (1, 96, 86,400 samples per day) and two space resolution levels (all aggregated, light and computer separated). Red and green sections show recognizable wasting and conservation behaviors, respectively. The dark gray areas indicate interpretable information. For instance, the red box [F] in (IV-2) shows the base power level of the computers.

In Figure 4, the power consumption plots are shown for six different combinations of time and space resolutions. If the power usage is measured only once per day for the aggregate usage of all lights, computers, and others in Table 1, the power consumption plot looks like (I). From this plot, we can tell the overall power usage level for each day, but probably that is all the information that can be extracted. In comparison, if the power usage is measured every 15 min (96 samples/day) and, furthermore, the lights and computers are measured separately, the resulting plots are much richer in information, as shown in (IV-1) and (IV-2). As an example, consider (IV-1), which is the aggregate for the usage of the lights only. The first red section, marked as [B] in (IV-1), indicates that all the lights were turned on together in the same 15 min interval in the morning. Considering that the graduate students do not have a fixed time schedule, and that it typically takes a while for the second student to show up in the office, this is undesired behavior. There is no need to turn on all the lights in an office that is used by 15~20 students when full. The second red section, [C] in (IV-1), indicates that the lights were never turned off during the night, another undesired behavior. The thin green section [D] in (IV-1) indicates that the lights were properly turned off the following night. The green section [E] in (IV-1) indicates that the light switches were sequentially turned on over several hours as more students arrived at the office. 

Clearly, a certain type of information cannot be obtained without increased time and space resolution. In Table 3, waste and conservation behaviors that can be detected with different levels of space and time resolution are summarized. For the time resolution, the same three levels as in Table 3 are used. For the computers, only three sensors were used to measure the power usage of 15~20 computers and many more monitors depending on their physical locations in the office. While this is a very high space resolution compared to most previous energy saving studies, it is worth noting that a sensor measurement per computer or a sensor measurement per person would have enabled a personalized waste investigation. When a person-level data collection was discussed, however, the participants expressed a very strong objection due to a possible compromise of privacy. In the study, we actually had four sensors (one for lights and three for computers), and therefore space resolution of four groups could have been utilized, but we focus on two groups (light and computer groups) because the additional information was marginally helpful in this study. 

During the pre-trial investigation, it became clear that the data quality in terms of time and space resolution was critical for identifying wasting and conservational behaviors. A high resolution of space-time data was essential for identifying the key behaviors and their relative importance for the site’s energy saving. In the following section, the actual energy saving experiments and what we learned from them are explained.

## 4. Experiments

To understand the effectiveness of data-driven analysis and to understand the maximum potential of energy saving without affecting normal activities in a campus environment, the experiment was designed to utilize the pre-trial data as much as possible and to aim for a maximum energy saving in one week. To make sure that the students were motivated and diligently made waste reduction efforts, a reward of a nice group dinner was promised, as long as the students saved a reasonably large amount of energy. The ‘large amount’ was not very clearly specified, because we wanted the students to make no less or no more than the best effort without sacrificing their normal activities; however, when pressured to specify the exact number, we indicated that a 20% saving would be considered to be more than successful.

**Figure 5:**
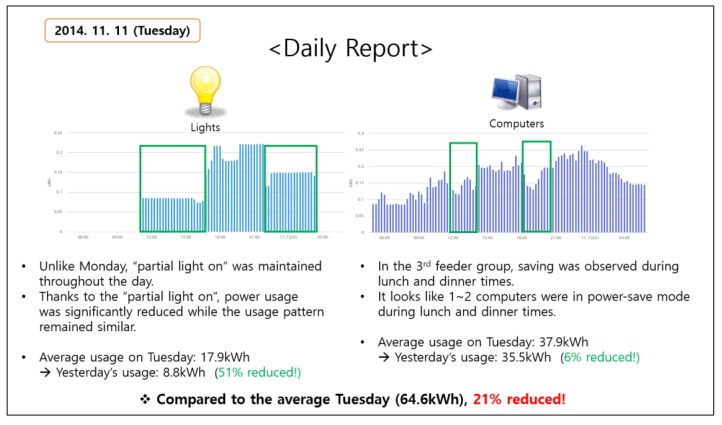
An example of the daily report delivered by the energy delegate (translated).

The experiment was designed to last for one week, with a dedicated energy delegate for close communication. The energy delegate visited the office every morning to talk with the students, and the delegate also delivered a daily report that contained the detailed information and instructions for energy saving (Figure 5). The report was generated by analyzing the previous day’s energy data. It contained a list of tips generated by inspecting the list of wasting behaviors explained in Section 4. For instance, if all the lights are simultaneously turned on by the first student at the office, the information was included in the report, and the energy delegate pin-pointed the behavior and asked for additional attention on the matter. The report also contained energy consumption information. Each day, students were informed whether they had successfully reduced power consumption on the previous day, and what % of reduction was achieved if they were successful. All the information was summarized in the daily report, which contained plots and texts. Overall, this experiment was an intensive energy saving exercise that was designed to last for only one week. 

### 4.1. Data-Driven Intervention Design

Findings from the pre-trial investigations utilizing high-quality data were fully reflected in the intervention design. Regarding the light usage behaviors, the list of interventions was ‘encourage partial light-on when possible’, ‘ask for full attention on preventing overnight light-on’, and ‘encourage lights and computers to be turned off during lunch time’. To assist the students, color stickers were deployed, and they visually showed the mappings between the switches and the corresponding light sections. This action was the direct result of pre-trial interviews, where students said it was cumbersome to try the switches until the desired light section only is turned on. 

Regarding the computer usage behavior, the most obvious problem was the students keeping computers on all the time. Proper settings of screensaver and sleep modes were strongly recommended, and the energy delegate provided support for configuration of computers whenever requested. As will be discussed later, it turned out that many of the students did not follow the recommendation, despite evident energy savings. Subsequent short interviews just after E1 revealed that students kept the computers on mainly to keep remote login enabled. In fact, they hardly used remote login, but they were simply concerned about rare but possible needs to connect to university network environment for free access to academic literature or concerned about occasional needs to open or transfer files to the office computers. Basically, keeping computers in sleep meant disabling remote login. This issue was identified only after experiment E1 and was addressed using WOL (Wake-On-Lan) in experiment E2. WOL is an Ethernet standard that allows a computer to be turned on or awakened by a network message called magic packet [32]. With WOL, students can keep the computers asleep without sacrificing the capability to awaken the computers and remotely connect. To make use of it, a low-cost WOL system had to be deployed in the office network (around 50 US dollars). Other than keeping computers and screens off when they are not being used, we did not pursue any computer related interventions.

To be sufficiently convinced that these interventions were likely to be effective to the experiment group, the quality of data had to be at least a medium level of time resolution (96 samples per day) and two groups of space resolution (light and computer) in Table 3. In reality, however, we freely investigated the high level of time resolution and four groups of space resolution. Some of the intervention designs and feedback generation, especially for computers, were possible only by inspecting the highest levels of time and space resolutions and additionally by interviewing the students.

### 4.2. Experiment E1

The first official experiment was launched on 10 November of 2014 and lasted for one week (see Table 2). The experiment proceeded smoothly in the beginning, but in a few days, it was noticed that the energy usage of light feeders was too low to be true. Upon further inspection, strip LEDs connected to an independent power source were discovered. It turned out that one of the students was too motivated and installed the strip LED lights. The experiment was immediately announced to be invalid, and the energy delegate stopped energy saving activities. 

Even though the experiment had to be abandoned, a post-analysis was performed to investigate energy saving behaviors related to computer usage. While there was a meaningful reduction according to the data analysis, some of the high-power computers were kept on during the night. The follow-up interviews revealed that many students still kept the computers on because of the remote-access concerns. Hence, the need for WOL was identified, and a WOL was installed before starting experiment E2. The use of WOL turned out to be very important, as will be explained in the next subsection.

### 4.3. Experiment E2

To make sure that we had a fresh and independent experiment, we waited for two months before starting the experiment E2. This time, the students were asked not to do anything abnormal or anything affecting their normal activities. Instead, they were asked to focus on the energy saving behaviors that we were suggesting. Additionally, we made sure that WOL was ready and easily usable. The energy delegate personally showed how easy it was to use the system, and provided personal support when requested. After the education, the experiment was executed for one week. As in experiment E1, daily reports were provided and the energy delegate diligently communicated with the students to encourage them. This time, the experiment was completed without any incident, and the resulting energy saving turned out to be more than 20%, as will be explained in Section 5.1.

## 5. Energy Saving Results

In this section, the energy saving results are provided based on power consumption analysis and data-driven energy saving behavior analysis. We also explain how the two methods are complementary to each other.

### 5.1. Power Consumption Analysis

**Figure 6:**
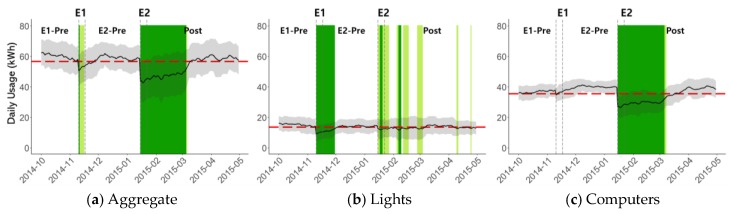
Power consumption analysis. The daily average power consumptions are shown. For color coding, the baseline (red dashed line) was set conservatively as the minimum observed value during the pre-trial period, and the energy saving of 5~10% is highlighted in light green, and 10% or above is highlighted in dark green. Aggregate includes lights, computers, and others in Table 1.

The daily average of power consumption is shown in Figure 6. The gray area indicates one standard deviation range. In each plot, the baseline consumption is shown together (red dashed line), where the baseline is defined as the minimum observed during E1-Pre. We deliberately chose the baseline to be very conservative, because the experiment size was not large enough to have a statistically stable control group, and because we wanted our analysis to be minimally susceptible to random factors. In terms of color coding, light green is used to indicate a 5~10% saving with respect to the baseline, and dark green is used to indicate more than a 10% saving. In Figure 6a, the result of the aggregate power usage is shown. Over 10% of reduction was achieved during the first few days of E1, but the saving became smaller after declaring the experiment to be invalid and dropped to less than 5% immediately after the 1-week experiment ended. As explained earlier, E1 was an interrupted experiment, and the students were asked to return to their normal behaviors. During E2, which was valid, over 10% of reduction (dark green) was achieved. In fact, the average energy saving with respect to the baseline was 25.4% during the experiment week (the 25.4% can be considered a reliable estimate of the true energy saving, because an abrupt drop in energy consumption can be observed in Figure 6a when E2 starts. None of the random factors showed such a strong influence throughout the entire data collection period, in which the data collection period included another 16 months after this work had been completed), and savings of over 10% lasted for another 44 days despite completely discontinued energy delegate activities and feedback. Considering that a new semester started in March and office and desk assignments were shuffled, the energy saving behaviors of E2 might have lasted longer than 44 days in the absence of the shuffling. Supporting evidence is that the dark green period suddenly ends with only two days of light green days in the end. This indicates that the energy saving behavior quickly and suddenly disappeared in early March. 

In Figure 6b, the result for the light group only is shown. During E1, the average was reduced because of the LED incident, but the effect disappeared within a few weeks of the experiment. During E2, the average was reduced, and the effect persisted mildly until the new semester shuffling. The amount of energy saving in terms of average power consumption, however, was relatively small compared to the computer group, and the color coding after E2 alternates among dark green, light green, and no color. In Figure 6c, the result for the computer group only is shown. The energy saving during E1 was negligible despite the energy delegate’s activities. The energy saving during E2 is significant, and the color coding is solid dark green until the new semester shuffling. Even though not shown in the plot, it was also found that the base power level was reduced significantly by utilizing WOL. Note that the large saving would have been impossible by just providing WOL only—it was essential to have a dedicated human delegate and a short-term incentive to motivate the students. Unlike the light group, the base power level was very large during E1-Pre, and the behavior change had a clear impact on base power. The peak power was also decreased considerably, indicating that not all the computers were simultaneously used even at the peak usage time. This result is consistent with the observation that the office occupancy by graduate students never reached close to 100%.

### 5.2. Pattern-Based Behavior Analysis

In the campus context, a variety of unpredictable factors can have a large influence on the power consumption. Some examples include exams, papers due, office allocation shuffling, graduation, and newcomers. Therefore, it is difficult to judge the intervention effectiveness unless there is a noticeably abrupt reduction in power consumption for a new intervention or a removed intervention. Even when such a reduction is clearly observable, as in the first day of E2 (see Figure 6a), it is not possible to be completely sure of the intervention effectiveness based only on power consumption analysis. The situation might not improve much even with a control group. The relative difference between a treatment group and a control group might not be trustable because of unpredictable factors that can cause each office’s power consumption to fluctuate independently. For instance, students in different offices typically prepare for different conferences with different deadlines. In fact, we had measurements from two extra offices but eventually decided not to use them as a control because there were too frequent fluctuations that were clearly specific to each office’s characteristics and activities. 

**Figure 7:**
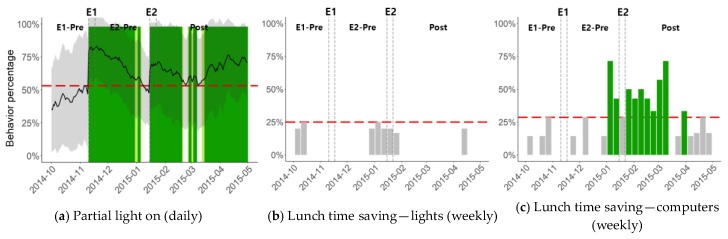
Pattern analysis of energy saving behaviors. Compared to the power consumption analysis, the absolute level of energy saving cannot be extracted, but the green areas guarantee that indeed energy saving behaviors have occurred more frequently. Therefore, pattern-based behavior analysis is an ideal complement to the power consumption analysis.

To overcome the limitation of analyzing power consumption curves only, we propose using data patterns that can be clearly mapped to known energy saving behaviors, are very unlikely to be observed by chance, and are very unlikely to be confused with activities that are irrelevant to energy saving behaviors. To show the effectiveness of this approach, we analyzed three of the items listed in Table 3, and the results are shown in Figure 7. In Figure 7a, the partial light-on ratio is shown where it is defined for each day as the percentage of light-on time with only 80% or fewer lights turned on. When this ratio is high, it indicates that the students frequently keep some of the unnecessary lights off. The resulting plot shows that partial light-on ratio was improved right after E1 and also after E2, confirming the behavior change. Interestingly, the behavior change was sustained for about two months after E1. Because the students were first educated on the matter during E1, it can be concluded that the energy saving behavior persisted for two months, whereas the power consumption plot in Figure 6b shows only 20 days of saving due to E1. It is also interesting to note that the partial light-on behavior was strengthened starting from 2015-03. It looks like the new students after office shuffling organically improved their behavior without any external intervention. 

In Figure 7b,c, lunch time saving results are shown for lights and computers. For each day, lunch-time saving behavior was examined by testing if the following equation was satisfied.

(Min power usage between 11:30 a.m. and 1:00 p.m.) < 0.85 × Min[(Max power usage between 10:30 a.m. and 11:30 a.m.), (Max power usage between 1:00 p.m. and 2:00 p.m.)](1)

If the condition is satisfied, it indicates that lunch time power usage was at least 15% less than before and after lunch time. Note that we chose 11:30 a.m. to 1:00 p.m. as lunch time in order to accommodate the broad spectrum of student behaviors in terms of when to have lunch. Using this definition, the percentage of days with lunch time saving behavior is shown for each week in Figure 7b,c. In Figure 7b, the result for lights is shown, indicating that lunch time saving hardly occurred before or after the interventions. This can be explained by the disparate lunch times of the students and indicates that interventions did not affect the lunch time saving of energy consumption for lights. In Figure 7c, the result for computers is shown. Unlike lights, lunch time saving was observed when E2 interventions were applied. The behavior was sustained for about two months, as shown in Figure 7c. It can be noted that the lunch time saving was increased even before E2 started, and the two green bars just before E2 are because of the early deployment of WOL solution and its use by the energy delegate for the training purpose. While students were not encouraged to start using WOL before E2, it looks like some of the students changed their computer setting or behavior before the intervention period started. For the E2 experiment week, the color code is actually grey and not dark green. Even though the color code is grey, the saving was above 25%, which can be considered to be high compared to the pre-trial period. As in Figure 6, the baselines (red dashed lines) of Figure 7 were set conservatively as the largest saving that was observed during the pre-trial period. For Figure 7c, perhaps the baseline is too conservative because the lunch time saving is either 0% or around 15% for most of the pre-experiment weeks.

## 6. Discussion

The energy saving potential in the university building was larger than expected. Overall reduction of 25.4% was achieved in E2 without sacrificing normal activities, and a significant portion of the saving was sustained for 44 days until the office and desk shuffling occurred. Overall, the large saving was possible mainly because of tailored interventions that were the direct outcomes of waste investigation using high-quality data.

The LED incident during E1 was completely unexpected. Despite the incident, a few key insights were identified from the experiment. As for the lights, pre-experiment investigation using high-resolution data was very useful. The tailored interventions derived from the findings were observable only with high-resolution data, and findings such as partial light-on and 24 h light-on were accurate and comprehensive enough to result in successful counseling to the students. When advised, the students quickly agreed and followed the instructions. As for the computers, pre-experiment investigation of high-resolution data was very helpful for identifying and analyzing the problems, including the severity of the always-power-on problem. Merely delivering the feedback and guidelines, however, was not sufficient to achieve the desired behavior change during E1. A further investigation beyond data analysis was necessary, and the main issue of ‘power-on for remote-access readiness’ was identifiable only after in-depth discussions between the energy delegate and the office students. 

While the data-driven pre-trial analysis was very helpful, the resulting interventions and advice were nothing really new. We neither discovered any new wasting behavior nor did we find a new way to reduce energy consumption. In this sense, it might be most accurate to say that the data-driven analysis was able to identify which known wasting behavior was occurring and how frequently. Such information was very helpful when the energy delegate tried to guide the students. When some of the students were in doubt, typically showing them the high-resolution data plots was sufficient to reach a quick agreement. Pin-pointing the list of saving items was important, as already known in the field [17], and the data-driven analysis was a pivotal element in our experiment. 

Besides the benefit of tailored intervention design, the data-driven analysis was shown to be effective for confidently detecting energy saving behaviors. Power consumptions of public spaces can be affected by many different factors [33], and even setting up a control group might not be sufficient to tell if energy saving truly happened. Therefore, utilizing data patterns and data-driven measures that are very unlikely to occur without serious effort in terms of energy saving can be a reliable way of confirming behavior changes. When there is no behavior change that can be detected, it might be reasonable to suspect whether or not the measured power consumption saving is indeed because of energy saving efforts. When both power consumption analysis and behavior detection results are positive, one can more reliably conclude a successful energy saving.

Furthermore, the detected energy saving behaviors can be used for generating highly effective feedback. Energy consumers usually do not understand what behavioral changes are needed in the local context [34]. The problem is aggravated when the consumers are not interested in energy saving, either. If high-resolution data can be collected and used for detecting energy saving behaviors and for pin-pointing the desired feedback [17], it can help improve the effectiveness of feedbacks and accuracy of influence assessments. Furthermore, such energy saving behavior detections can be used to evaluate whether a group of users is making more effort than another group, even when a direct comparison of quantitative consumption amounts is deemed inappropriate. Therefore, the behavior detection using high-resolution data may be a viable solution for performing a large-scale experiment that includes many offices with heterogeneous characteristics.

Whereas the scope of this paper is limited to lights and computers, data of HVAC was also available. The amount of energy saving on HVAC was comparable to the savings of lights and computers together, but the result is excluded in this paper because there was a central heating system during daytime and students kept the local HVAC completely off during the E2 period. There was no way to tell if the non-use of local HVAC was caused by the intervention or not, and the weather became warm after E2, thus making it difficult to conclude whether or not there was a behavior change.

A 25.4% energy saving potential was identified in the experiment, but the saving was achieved at the cost of a dedicated human delegate and a short-term incentive. To overcome this limitation, we actually conducted an expanded experiment after the study was completed, and the expanded experiment aimed at sustainability with minimal use of resources. Hence, the energy delegate was removed, and only wall-mounted dashboard screens in the office were allowed. The dashboards were the main channel for intervention, and a cost-effective incentive was introduced only at the last stage of the experiment. We have experimented in many different ways for 34 weeks concurrently over three offices and acquired a rich set of interesting data. In the end, however, we decided to abandon the entire second experiment result, because there were too many factors that were specific to each office’s characteristics and activities, and because the longevity of the experiment allowed even more random factors to be part of the data. Despite the high-resolution data and its tremendous benefits, we were not able to derive sufficiently rigorous quantitative conclusions in the campus environment. In fact, we believe our experiment environment was one of the most challenging, and that the results in this work were possible only because of the advantage of high-resolution data and the relevant data-driven approaches. For less challenging building environments with fewer random factors, it would be much less difficult to perform a similar experiment and quantitatively evaluate the energy saving amount. In general, we believe the findings in this work will be useful for any environment, but some investigations and customizations might be necessary to identify how and what parts of insights to utilize for the given environment.

## 7. Conclusions

With the advancement of sensor technology and the spread of cost-efficient energy data platforms, it is time to consider how data-driven approaches can be better integrated into energy saving activities and beyond. In this work, we have focused on the benefit of collecting high-resolution data and utilizing them for intervention design and behavior detection. With a data-driven pre-trial analysis, we were able to achieve 25.4% of energy reduction in a campus environment within a week. The reduction was sustained for 44 days without any further intervention. The quantitative analysis, however, could be vulnerable to uncontrollable factors that are abundant in a campus environment. To address this issue, we showed that pattern detections using high-resolution data can better confirm the actual change in behaviors because energy saving behaviors show distinct patterns and are robust against the random factors. The detected energy saving behaviors are expected to be useful for pin-pointing the desired energy saving actions and for comparing the effectiveness of interventions among groups with heterogeneous characteristics. As seen in this work, the value of high-quality data and data-driven approaches needs to be carefully considered in modern energy saving and other energy activities. 

## Figures and Tables

**Table 1:** Monthly consumptions averaged over two months before experiments (2014.9.1~2014.10.31).

Light	Computer	Others	Total
444 kWh	1055 kWh	271 kWh	1770 kWh
25%	60%	15%	100%

**Table 2:** Timeline of the experiments.

Name	Period (Duration)	Feedback	Other Supports
**E1-Pre**	2014.10.01~2014.11.09 (6 weeks)	-	-
**E1**	2014.11.10~16 (1 week)	Daily report, Energy delegate	Light switch sticker Incentive,
**E2-Pre**	2014.11.17~2015.1.14 (9 weeks)	-	-
**E2**	2015.1.15~21 (1 week)	Daily report, Energy delegate	Light switch sticker Incentive, Wake-on-Lan
**Post**	2015.1.22~2015.4.30 (8 weeks)	-	Light switch sticker Wake-on-Lan

**Table 3:** Waste and conservation behaviors that can be detected depending on the data resolution.

		Space Resolution	
		1 Group (Aggregate)	2 Groups (Light & Computer)
**Time Resolution**	Low (1-sample/day)	For each day, What is the aggregate power usage?	For each day, What is the % of power spent on lights? What is the % of power spent on computers?
	Medium (96-samples/day)	What is the base power usage? ([A] in Figure 4)	Light Does the first person to the office turn on all lights or only the necessary lights? ([B], [E] in Figure 4) Does an unnecessary overnight light-on occur? ([C] in Figure 4) Does the last person leaving the office turn off the lights? ([D] in Figure 4) Are lights turned off during lunch time? Computer What is the base power level? ([F] in Figure 4) What is the ratio between base power level and peak power level? Is computer usage reduced during lunch time?
	High (86400-samples/day)	Exactly when do power-on and power-off events occur?	Light How many light switches are simultaneously turned on during a ‘power-on’ event? ([G] in Figure 4) Computer Exactly when do computer-on and computer-off events occur? ([H]: two on events, [I]: one off event) How many computer-on/off events occur in a day?
